# Dental and Orthopaedic Implant Loosening: Overlap in Gene Expression Regulation

**DOI:** 10.3389/fimmu.2022.820843

**Published:** 2022-02-11

**Authors:** Sabine Schluessel, Eliza S. Hartmann, Miriam I. Koehler, Felicitas Beck, Julia I. Redeker, Maximilian M. Saller, Elif Akova, Stefan Krebs, Boris M. Holzapfel, Susanne Mayer-Wagner

**Affiliations:** ^1^ Department of Orthopaedics and Trauma Surgery, Musculoskeletal University Center Munich (MUM), University Hospital, Ludwig Maximilian University (LMU) Munich, Munich, Germany; ^2^ Gene Center, Laboratory for Functional Genome Analysis, University Hospital, Ludwig Maximilian University (LMU) Munich, Munich, Germany

**Keywords:** dental and orthopaedic implant failure, osteoclastogenic regulation, periprosthetic tissue, immune reaction, RNA-sequencing, immune osteoclastic cells

## Abstract

**Objectives:**

Endoprosthetic loosening still plays a major role in orthopaedic and dental surgery and includes various cellular immune processes within peri-implant tissues. Although the dental and orthopaedic processes vary in certain parts, the clinical question arises whether there are common immune regulators of implant loosening. Analyzing the key gene expressions common to both processes reveals the mechanisms of osteoclastogenesis within periprosthetic tissues of orthopaedic and dental origin.

**Methods:**

Donor peripheral blood mononuclear cells (PBMCs) and intraoperatively obtained periprosthetic fibroblast-like cells (PPFs) were (co-)cultured with [± macrophage-colony stimulating factor (MCSF) and Receptor Activator of NF-κB ligand (RANKL)] in transwell and monolayer culture systems and examined for osteoclastogenic regulations [MCSF, RANKL, osteoprotegerin (OPG), and tumor necrosis factor alpha (TNFα)] as well as the ability of bone resorption. Sequencing analysis compared dental and orthopaedic (co-)cultures.

**Results:**

Monolayer co-cultures of both origins expressed high levels of OPG, resulting in inhibition of osteolysis shown by resorption assay on dentin. The high OPG-expression, low RANKL/OPG ratios and a resulting inhibition of osteolysis were displayed by dental and orthopaedic PPFs in monolayer even in the presence of MCSF and RANKL, acting as osteoprotective and immunoregulatory cells. The osteoprotective function was only observed in monolayer cultures of dental and orthopaedic periprosthetic cells and downregulated in the transwell system. In transwell co-cultures of PBMCs/PPFs profound changes of gene expression, with a significant decrease of OPG (20-fold dental versus 100 fold orthopaedic), were identified. Within transwell cultures, which offer more *in vivo* like conditions, RANKL/OPG ratios displayed similar high levels to the original periprosthetic tissue. For dental and orthopaedic implant loosening, overlapping findings in principal component and heatmap analysis were identified.

**Conclusions:**

Thus, periprosthetic osteoclastogenesis may be a correlating immune process in orthopaedic and dental implant failure leading to comparable reactions with regard to osteoclast formation. The transwell cultures system may provide an *in vivo* like model for the exploration of orthopaedic and dental implant loosening.

## 1 Background

The initial triggers of orthopaedic and dental implant loosening differ at first glance significantly also due to the aberrant microbiological environment. However, in both conditions the formation of a fibrous peri-implant tissue is initiated. Although it might be assumed that fundamentally different loosening processes and immune regulations occur in orthopaedic and dental implants, similar cytokines are involved in cascades of both processes, which lead to the formation and activation of osteoclasts. Both peri-implant tissues of loosened endoprostheses consist mainly of macrophages and periprosthetic fibroblast-like-cells (PPFs) (1).

PPFs in orthopaedic peri-implant tissues express TNFα, a cytokine that is an important signaling metabolite for local and systemic inflammatory reactions (2, 3). When MCSF is added *in vitro*, TNFα-expressing PPFs cause increased osteoclast formation and thus might contribute to endoprosthetic loosening (2, 3). TNFα is also highly expressed in peri-implant tissues of loosened dental implants (4). In gingival fibroblasts, TNFα leads to the release of prostaglandin E2 (PGE2) (5). PGE2, a signal protein of bone resorption, has been detected in orthopaedic and dental peri-implant tissues (6, 7). PGE2 synthesizing cyclooxygenase-2 (COX-2) is expressed in PPFs and stimulated by titanium particles (8). PGE2 induces an increased expression of Receptor Activator of NF-κB ligand (RANKL) in PPFs (9). RANKL has a strong impact on the regulation of bone formation and resorption. RANKL activates osteoclasts by binding to the RANK receptor of osteoclast precursor cells, thereby inducing their differentiation into osteoclasts. RANKL expression of PPFs thus directly induces osteoclast formation in orthopaedic and dental peri-implant tissues (10–12).

The opponent Osteoprotegerin (OPG) acts as decoy receptor for RANKL and inhibits osteoclast differentiation. In periodontitis, an increased RANKL/OPG Ratio (up-regulation of RANKL and down-regulation of OPG) is described (13).

PPFs also express matrix metalloproteinases (MMPs), which are found in elevated concentrations in orthopaedic and dental peri-implant tissues (14, 15). MMP13 degrades bone due to its substrate specificity for collagen type 1 (16). The increased expression of MMPs leads to collagen degradation in peri-implantation tissues. In addition, MMPs are found in periosteoclastic cells (15) and in subosteoclastic resorption lagoons of osteoclasts, thereby contributing to further bone loss (17). Due to their expression patterns, PPFs in peri-implant tissue assume similar functions to so-called “aggressive” fibroblasts in rheumatoid arthritis (18).

Osteocalcin (OCN), an extracellular matrix protein, is synthesized by mature osteoblasts and is able to influence bone mineralization and remodeling (19). Cathepsin K (CTSK), a cysteine protease, is mainly expressed by osteoclasts and is involved in collagen cleavage in the extracellular matrix (20). Increased levels of OCN and CTSK were found in peri-implant crevicular fluid and might indicate a higher bone turnover in implants (20, 21). Mandelin et al. showed that interface tissue fibroblasts are also able to secrete CTSK (10).

Tartrate-resistant-acid-phosphatase (TRAP) is an osteoclast-specific marker closely linked to bone resorption. In early phases of orthopaedic implant loosening increased TRAP levels are described, while late phases correlate with decreased amounts of TRAP (22).

These examples indicate that similar cytokines, prostaglandins and MMPs are involved in orthopaedic and dental peri-implant tissues that contribute to the formation and activation of osteoclasts.

Whether the processes in oral and orthopaedic peri-implant tissues might be closer related, and an overreaction of the immune system has a high impact on marginal bone loss and failure of dental and orthopaedic implants is discussed by Albrektsson et al. (23). The foreign body reaction might play an important part in oral as well as orthopaedic implants.

In order to examine the overlying immune effects with regards to osteoclast formation of dental and orthopaedic periprosthetic tissues, a common model of co-cultures, containing of peripheral blood mononuclear cells (PBMCs) and PPFs, was used (24, 25).

As *in vitro* cell studies often lack the complex three-dimensional component, a multilayer transwell (TW) culture system was applied. TW cultures have the advantage to provide an optimal medium supply from two sides improving intercellular connections and direct cell-cell contacts (26).

TW cultures have been described to increase the number of cells in fibroblast cultures (27) and changes expression patterns of co-cultured orthopaedic PPFs (28). The effect of transwell cultures to modify the expression of major mediators of osteoclastogenesis was proven for PPF cells from orthopaedic implants (28).

To our knowledge, there have not been any studies comparing periprosthetic tissues of dental and orthopaedic cells. The hypothesis of overlapping peri-implant tissue reactions in dental and orthopaedic implant failure was investigated within this study using a co-culture model of periprosthetic fibroblast like cells from orthopaedic and dental implants and examining their effect on immune cells like PBMCs in terms of osteoclastogenesis and bone loss.

## 2 Material & Methods

### 2.1 Patients

Peri-implant tissues were collected from eight patients (six female, two male; mean age 64, age range: 45 to 76 years) undergoing dental implant revision due to aseptic peri-implantitis. The diagnosis was conducted by the attending implantologist using the established criteria of the Sixth European Workshop on Periodontology (29, 30). Patients with allergies to components of the endoprosthetic material, early implant failure (<12 months), disorders of bone metabolism, rheumatoid arthritis or other inflammatory arthritis were excluded. Tissue samples were immediately incubated in the operating room in Dulbecco´s Modified Eagle Medium (DMEM; Biochrom, Berlin, Germany) with 60 IU/ml penicillin, 60 µg/ml streptomycin (Biochrom, Berlin, Germany) and 0.25 µg/ml Amphotericin B (Sigma–Aldrich Co., St. Louis, MO, USA). The study was approved by the medical ethics committee of the Ludwig-Maximilians-University Munich, Germany. Based on the design of the study using disposable material no patient consent was required. The experiment was carried out three times.

### 2.2 Isolation of Fibroblast-Like-Cells

Following the protocol of Hartmann et al. (31), collected tissue was washed with phosphate-buffered saline (PBS) (Biochrome, Berlin, Germany), cut into 2 mm sized pieces and digested with Dulbecco´s Minimal Essential Medium (DMEM, Biochrom, Berlin, Germany) containing 1mg/ml collagenase Type 1 (Sigma-Aldrich Co., St. Louis, MO, USA) for 30 min at 37°C. Next, a second digestion step with Versene (Invitrogen, Darmstadt, Germany) for 60 min at 37°C was conducted. After the digestion, the remnant was sterile-filtered using a 70 μm cell strainer (BD Bioscience, San Jose, USA) and centrifuged for 5 min at 1500 rpm. The pellet was resuspended in DMEM supplemented with 10% fetal bovine serum (FBS; PAA Laboratories, Cölbe, Germany), 2 mM L-glutamine, 60 IU/mL penicillin, 60 µg/mL streptomycin (all Biochrom, Berlin, Germany), 0.075 µg/mL amphotericin B (Sigma-Aldrich Co., St. Louis, MO, USA), 5 ml non-essential amino acids (50x, Thermo Fisher, New York, NY, USA) and cultured in a T75 culture flask (Nunc, Roskilde, Denmark) at a density of 3.5 x 10^3^/cm^2^ at 37°C and 5% CO_2_. During the initial seven days, FBS was increased to 20%. The medium was changed twice a week. Cells were passaged at 80-90% confluence by using 0.05% trypsin (Biochrom Berlin, Germany) containing 0.02% ethylenediaminetetraacetic acid (EDTA, Biochrom Berlin, Germany).

Cultures of fibroblast-like cells were assessed histochemically for the absence of TRAP by using a TRAP detection kit (Sigma-Aldrich Co., St. Louis, MO, USA) to exclude the presence of TRAP positive cells, which would have falsified controls (2, 31, 32). Cell cultures were also tested for mycoplasma contamination in passage one performing PCR Mycoplasma Test Kit I/C (PromoCell, Heidelberg, Germany). The cells were used at passage three for the following monolayer and transwell culture experiments.

Additionally, primary tissues (n=8) were used for RNA isolation in order to compare the results to *in vivo* conditions (=baseline). Therefore, these tissues were directly placed in RNA later (Sigma-Aldrich Co., USA). The next day RNA later was removed and tissues stored at -80°C.

### 2.3 Isolation of PBMCs

Buffy coats (n = 4, male donors, blood group A (2 times), B and 0 respectively, all rhesus positive) were received from the German Red Cross Blood Donor Service at the university of Ulm, Germany. Buffy coats were processed on the same day following the established protocol (33).

### 2.4 Cell Culture Experiments

Cells were cultured following a protocol published by Koehler et al., 2019 on conventional 24-well monolayer (ML) plates for adherent cells (Nunc, Roskilde, Denmark) and on the membranes of 24-transwell (TW) plate inserts (pore size 0.4 µm, Nunc, Roskilde, Denmark). Cell culture experiments were performed in ten groups (please see **Table 1**). In both culture types a cell density of 1.2 x10^5^ PPFs and 6x10^6^ PBMCs per well was used. The PBMCs were seeded on day 0, followed by medium change on day 1 in order to remove non-adherent cells. On day 3, PPFs were added to co-cultures. The same medium was used for all groups containing α-Minimal Essential Medium (α-MEM, Biochrom, Berlin, Germany), 10% fetal bovine serum (FBS; PAA Laboratories, Cölbe, Germany), 2 mM L-glutamine, 60 IU/mL penicillin, 60 µg/mL streptomycin (all Biochrom, Berlin, Germany) and 0.075 µg/mL amphotericin B (Sigma-Aldrich Co., St. Louis, MO, USA). Cells were cultivated at 37°C and 5% CO2. The medium was changed three times a week.

**Table 1:** Cell culture groups.

Cell type		Cell culture	Stimulated with RANKL; MCSF
1	PPFs (negative osteoclastic control)	ML	–
2	PBMCs (positive osteoclastic control)	ML	+
3	PBMCs	ML	–
4	co-culture (PPFs and PBMCs)	ML	–
5	co-culture (PPFs and PBMCs) *	ML	+
6	PPFs (negative osteoclastic control)	TW	–
7	PBMCs (positive osteoclastic control)	TW	+
8	PBMCs	TW	–
9	co-culture (PPFs and PBMCs)	TW	–
10	co-culture (PPFs and PBMCs) *	TW	+

*Only performed on dentin chips; ML, monolayer; TW, transwel.

In order to avoid bias by donor-specific cell characteristics, PPFs were separately co-cultivated with PBMCs of two different donors (donor of PBMCs =D). The stimulation of PBMCs (25 ng/ml MCSF (recombinant human MCSF, R&D Systems, Minneapolis, MN, USA) took place on day 0, 1 and 3 and with RANKL (recombinant human sRANK Ligand, Peprotech, Rocky Hill, NJ, USA) which started on day 6 with 10 ng/ml and was increased to 20ng/ml on day 8.

### 2.5 Hoechst and TRAP Staining of Monolayer Cultures

The staining of TRAP was performed in conventional 24-well plates by using the commercial kit (Sigma-Aldrich Co., St Louis, MO, USA) on day 28 as recommended by the manufacturer. After rinsing, cell cultures were incubated with Hoechst Solution (Invitrogen, Darmstadt, Germany 1:1000 PBS) for 10 min at RT in the dark. The presence of multinucleated TRAP positive cells was detected by light microscope (Axiovert 40, Zeiss, Germany) and fluorescence microscope (BZ9000, Keyence, Japan).

### 2.6 Dentin Assay

In order to investigate osteoclastogenesis *via* bone resorption cells were cultivated on dentin chips as described by Koehler et al. (28). On day 29, the dentin chips were stained with Hoechst, as described. Afterwards, cells were removed by adding sodium hypochlorite (Merck, Darmstadt, Germany). After rinsing and cleaning in 80% ethanol, the slices were stained with 1% toluidine blue solution (Waldeck, Münster, Germany) for 10 seconds until they appeared in blue color. After rinsing the chips again with tap water, dentin chips surfaces were scanned for resorption lacunae (BioRevo Fluorescence Microscope, Keyence, Neu-Isenburg, Germany). The staining and dentin assay was carried out three times.

### 2.7 Quantitative Real-Time PCR (qRT-PCR) of Monolayer and Transwell Cultures

RNA was isolated from monolayer and transwell cultures on day 0, 13 and 20 following the protocol of Koehler et al. (28). For cDNA synthesis, 0.5 µg RNA was reversed-transcripted using QuantiTect Reverse Transcription Kit (Quiagen, Hilden, Germany). For qRT-PCR a Light Cycler (LightCycler 96 Real-Time PCR System, Roche Diagnostics, Mannheim, Germany) was used. Gene expression analysis of the following markers was implemented: *Elongation factor 1alpha (EF1a*, housekeeping gene), *CTSK*, *MCSF*, *TNFα*, *RANKL*, *RANK*, *OPG*, *OCN* and *TRAP*. Amplification reactions were performed using Light cycler^®^ Fast Start Essential DNA Master Kit (Roche): 5 μL of FastStart Essential DNA Green Master Mix (Roche Diagnostics, Mannheim, Germany), 2.5 μl of 1:3 diluted cDNA and 0.3 μl (300 nM)/0.5 μl (500 nM) of primer were used, adding PCR grade water until reaching a total volume of 10 μl. Time, temperature and concentration of each primer are shown in **Table 2**. Reactions were performed in triplicates. For the relative quantification the 2^-DCT^ method was used, to provide comparison between gene expression levels of different genes. The 2^-DCT^ method was chosen to keep the comparability of expression levels of *RANKL* and *OPG*, which would have been lost using the 2^-DDCT^ method.

**Table 2:** Primers for quantitative real-time PCR.

Gene	Primer Sequences (5´– 3´)	Primer Concentration (n)	Annealing Temperature (AT)	Amplification (95°C - AT - 72°C)	Ampli-con size (bp)
**CTSK** (31, 34)	TTCCCGCAGTAATGACACC TTTCCCCAGTTTTCTCCCC	500 nM	63°C	10 s - 10 s - 20 s	615
**EF1a** (28)	AGCGCCGGCTATGCCCCTG CTGAACCATCCAGGCCAAAT	300 nM	60°C	15 s - 60 s - 10 s	59
**MCSF** (28, 34)	CCGAGGAGGTGTCGGAGTAC AATTTGGCACGAGGTCTCCAT	300 nM	60°C	10 s - 10 s - 15 s	100
**OCN**	TGAGAGCCCTCACACTCCTC ACCTTTGCTGGACTCTGCAC	500 nM	60°C	10 s - 10 s - 15 s	209
**OPG** (28, 34)	CTGCGCGCTCGTGTTTC ACAGCTGATGAGAGGTTTCTTCGT	300 nM	60°C	30 s - 60 s - 15 s	100
**RANK** (31)	CCTGGACCAACTGTACCTTCCT ACCGCATCGGATTTCTCTGT	300 nM	60°C	10 s - 10 s - 15 s	67
**RANKL** (28, 34)	CATCCCATCTGGTTCCCATAA GCCCAACCCCGATCATG	300 nM	60°C	10 s - 10 s - 15 s	60
**TNFα** (28, 34)	CCCAGGGACCTCTCTCTAATC GCTTGAGGGTTTGCTACAACATG	300 nM	60°C	30 s - 60 s - 15 s	103
**TRAP** (31)	TAGCCGGAAACCATGACCACC GATGCCCACGCCATTCTCATC	500 nM	65°C	10 s - 10 s - 15 s	446

Forward and reverse primers for quantitative real-time PCR.

### 2.8 RNA-Sequencing and Analysis of Monolayer Cultures

Total RNA from monolayer cultures of PPFs, PBMCs, and co-cultures on day 0, 13 and 20 was isolated with the Trizol method. RNA-seq libraries were generated from 200 ng of total RNA using the mRNA SENSE kit (Lexogen, Vienna, Austria) according to the manufacturer’s protocol. Multiplexed libraries were quality controlled on Agilent Bioanalyzer, pooled in equimolar amounts and sequenced in 100 bp single read mode on an Illumina HiSeq1500 instrument (Illumina, San Diego, CA, USA). Fastq files were demultiplexed according to the barcodes used for generation of each sample. Reads were aligned to the human genome (release GRCh38.101) using STAR (version 2.7.2b). Low gene expressions were filtered out by minimum 10 reads per gene cut off and 26,382 genes remained for further analysis. Normalization performed through variance stabilizing transformation (vst) for Principal Component Analysis (PCA). Top 50 differentially expressed genes defined through vst expression variance between each group. Significant differential gene expression was analyzed using DESeq2 (version 1.28.1) with 0.05 p-adjusted value cut off and 2 and -2 Log2FoldChange cut off for each group comparison. From 26,382 genes, 567 genes were differentially expressed between dental and orthopaedic co-cultures significantly. Furthermore, significant genes defined by the DESeq2 analysis of mono and co-cultures, independent from the derived location, were used to define significant Gene Ontology Biological Pathways by clusterProfiler (version 3.14) (35).

### 2.9 Statistical Analysis

Graph Pad Prism 8.3.0. for Windows (GraphPad Software, San Diego, CA, USA) was utilized to analyze the data. For significance testing t-tests for unpaired samples were used. A p-value < 0.05 was considered significant.

## 3 Results

General remark: Staining, Resorption Assay and RT-PCR results are only presented for dental cultures. The results of the orthopaedic cultures were already published 2019 by Koehler et al. (28). The Sequencing data shows the results for dental and orthopaedic monolayer cultures.

### 3.1 TRAP Staining Was Detected in Stimulated and Unstimulated PBMCs and in the Co-Culture Groups

**Figure 1:**
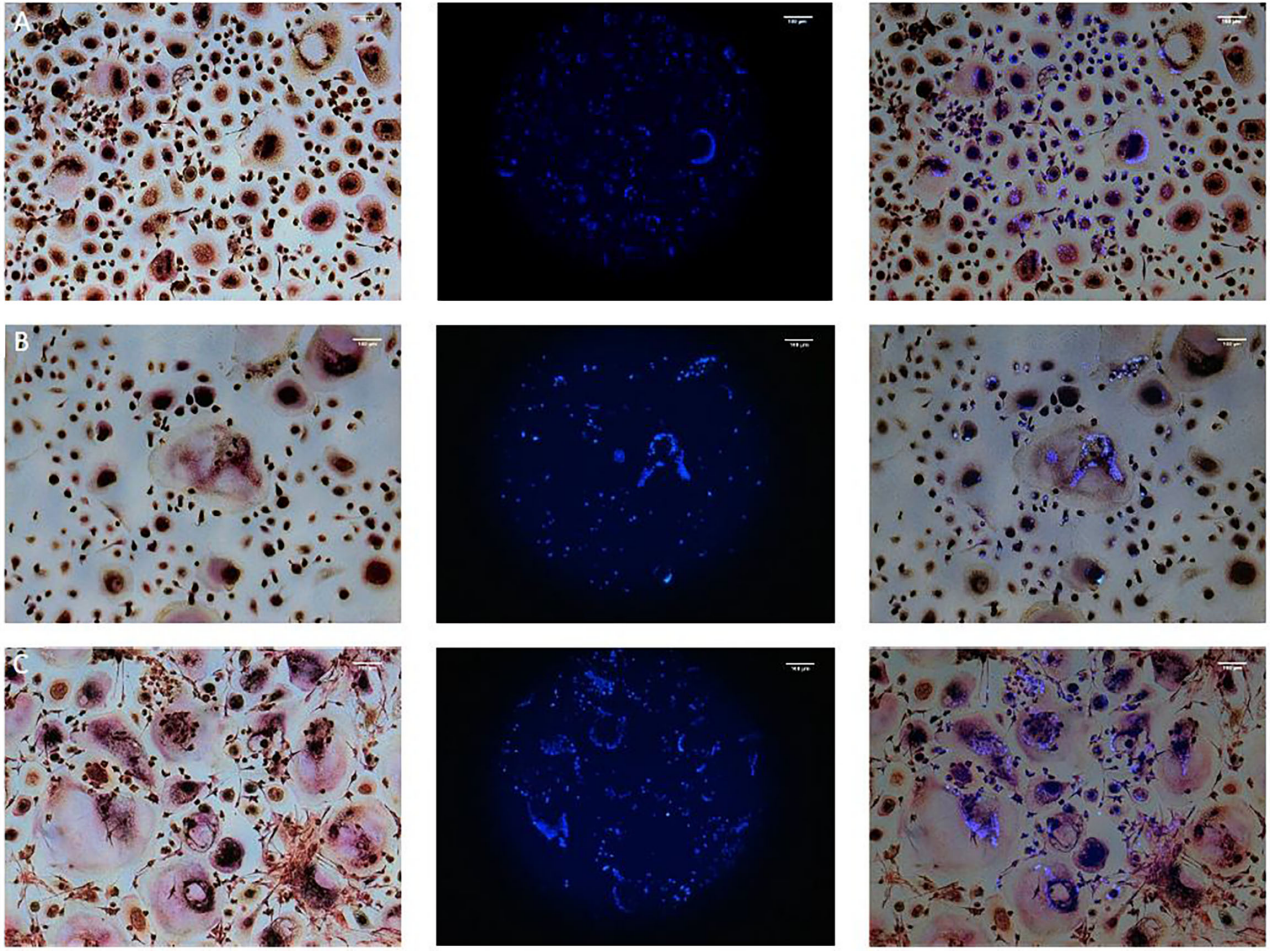
TRAP and Hoechst staining on day 28. Monocultures of PBMCs cultivated with **(A)** and without **(B)** additional M-CSF/RANKL and **(C)** Co-cultures of PBMCs/PPFs. Different sizes of multiple TRAP positive multinucleated cells in **(C)** additionally surrounded by small spindle-shaped cells. Photos were taken via light microscope (left), a fluorescence filter (middle), and combined (right). Scale bar = 100 μm.

TRAP staining enables the detection of multinucleated osteoclast like cells, but is no specific marker of osteoclastogenesis like bone resorption. All monocultures of PPFs showed TRAP negative, mononuclear, spindle-shaped cells. Mono- and co-cultures of PBMCs all contained TRAP positive multinucleated cells of various sizes with around 5 nuclei (**Figures 1A** **–C**). **Figure 1** shows PBMCs cultivated with (**Figure 1A**) and without (**Figure 1B**) additional M-CSF/RANKL. Co-cultures of PBMCs/PPFs (**Figure 1C**) also showed multiple TRAP positive multinucleated cells. In this study, the experiments showed TRAP signals in stimulated and unstimulated PBMCs and in the co-culture group. The size of the multinucleated cells was the main difference between groups. Visually, co-culturing led to larger multinucleated cells, surrounded by spindle shaped fibroblast-like cells.

### 3.2 Only Stimulated PBMCs From Monolayer and Transwell Cultures Showed Resorption Activity on Dentin Chips

**Figure 2:**
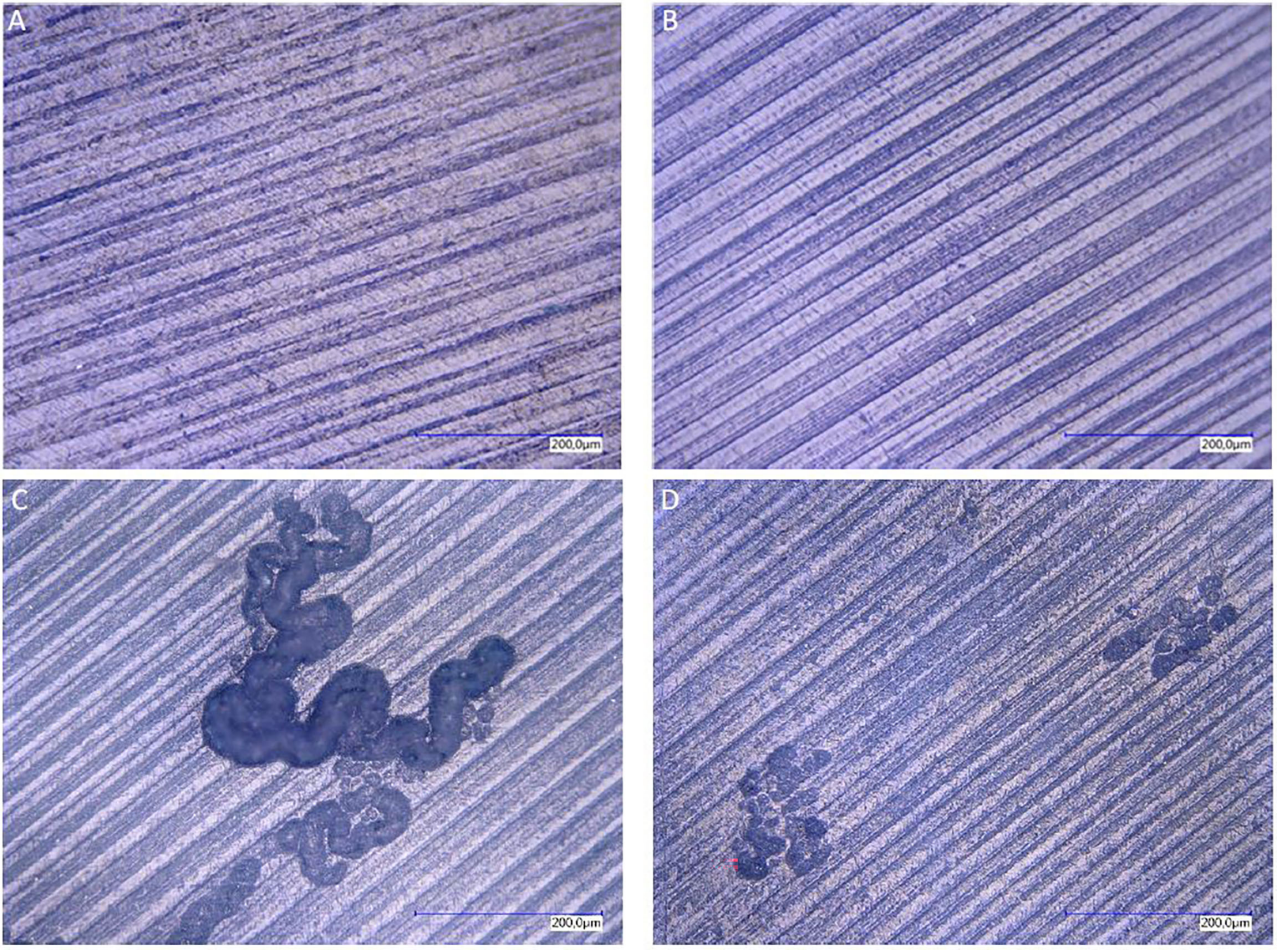
Resorption lacunae on dentin after staining with toluidine blue. Resorption lacunae on dentin after staining with toluidine blue. Lack of resorption pits in monolayer PBMC/PPFs co-cultures **(A)**, even when cultivated in the presence of additional MCSF and RANKL **(B)**. PBMC monocultures stimulated with MCSF and RANKL, cultivated in monolayer **(C)** or on transwell membranes [and transferred on dentin **(D)**], showed traces of osteolysis.

Resorption assay on dentin chips was used as gold standard to proof complete osteoclastogenesis on day 29. Positive controls of PBMCs cultivated with MCSF and RANKL from monolayer and transwell cultures showed both resorption activity on dentin chips. In general, resorption lacunae of these monolayer cultures were significantly larger than those of transwell PBMC monocultures (**Figures 2C, D**). All monocultures of unstimulated PBMCs did not show any sign of osteolysis, although they contained TRAP positive multinucleated cells.

No resorption lacunae were found on dentin chips of unstimulated PPF/PBMC co-cultures (monolayers and transwells). Stimulated PPF/PBMC co-cultures also showed no signs of osteolytic lacunae (**Figures 2A, B**). Monocultures of PPFs showed no resorption lacunae. (The presence of cells on the dentin chips was proven before with Hoechst staining *via* fluorescence filter. The presence of single nuclei indicated PPFs, while several nuclei in one space are typical for PBMCs (**Figures 1A** **–C**).

### 3.3 Quantitative Real Time PCR

Gene expressions of *MCSF*, *RANKL*, *RANK*, *OPG*, *TNFα*, *OCN*, *CTSK* and *TRAP* from PPF, PBMC and PPF/PBMC co-cultures were determined on day 0, 13 and 20 from monolayer and transwell plates.

#### 3.3.1 Monolayer Co-Cultures Show High Levels of OPG

**Figure 3:**
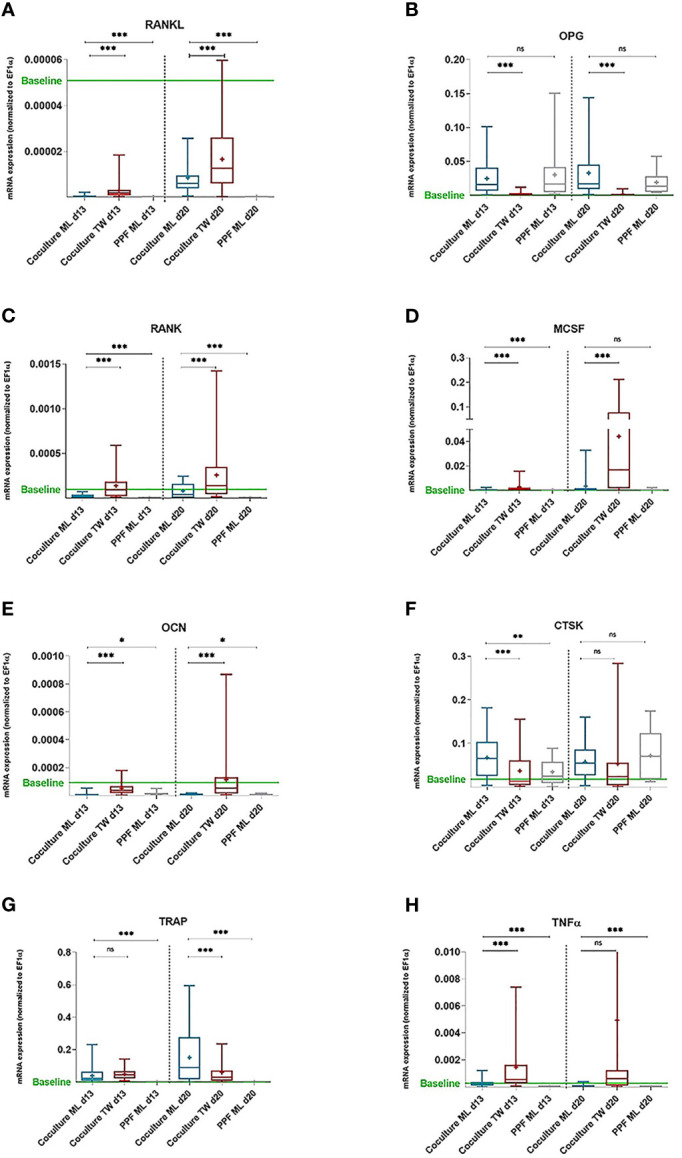
mRNA expression of PPF monocultures and PBMC/PPF co-cultures (day 13 and 20). mRNA expression of PPF monocultures and PBMC/PPF co-cultures. Relative mRNA Relative mRNA expression **(A)** RANKL, **(B)** OPG, **(C)** RANK, **(D)** MCSF, **(E)** OCN, **(F)** CTSK, **(G)** TRAP and **(H)** TNFa (normalized to housekeeping gene EF1a) in co-cultures of PPF and PBMCs in monolayer (ML, n = 16) and transwell (TW, n = 16) as well as in monocultures of PPF in monolayer (n = 8) on day 13 and day 20. Bands inside the boxes indicate group medians,crosses indicate group means. End of whiskers represent minimum and maximum values. Baseline refers to relative mRNA expression of original periosteolytic tissue (n=8). p values are indicated with * (p ≤ 0.05), ** (p ≤ 0.01),*** (p ≤ 0.001), ns, not significant.

**Figure 4:**
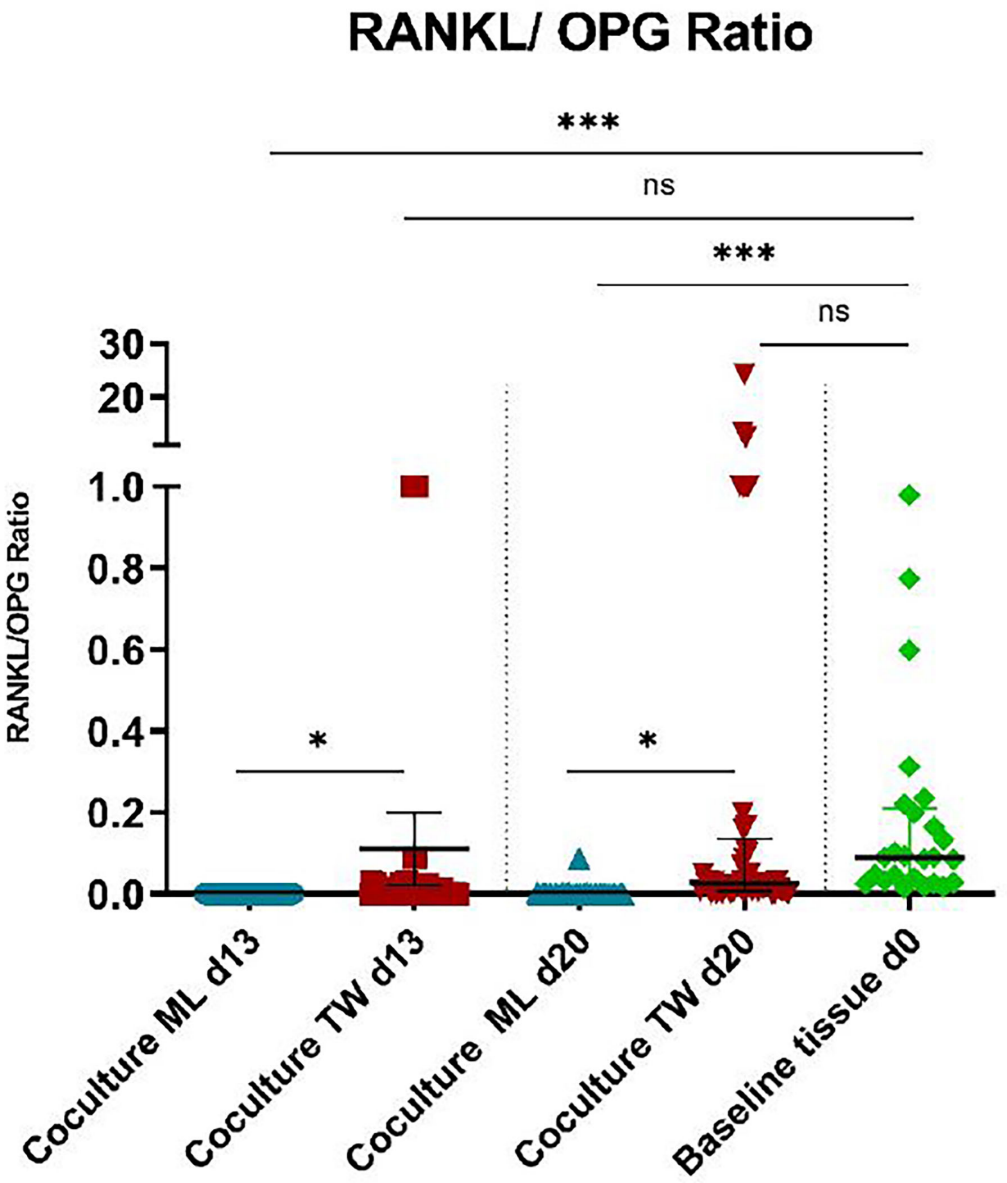
RANKL/OPG ratios of PBMC/PPF co-cultures and baseline tissue (day 0). * (p ≤ 0.05), *** (p ≤ 0.001), ns, not significant.

When compared to negative control (=monolayer PPF cultures), monolayer PPF/PBMC co-cultures showed mostly elevated expression levels for *RANKL* (**Figure 3A**, d13: p<0.001, d20: p<0.001), *RANK* (**Figure 3C**, d13: p<0.001, d20: p<0.001), *MCSF* (**Figure 3D**, d13: p<0.001, d20: p=0.940), *OCN* (**Figure 3E**, d13: p= 0.0421, d20: p=0.0325), CTSK (**Figure 3F**, d13: p=0.0012 d20: p=0.1855), *TRAP* (**Figure 3G**, d13: p<0.001, d20: p<0.001) and *TNFα* (**Figure 3H**, d13: p<0.001, d20: p<0.001). There was no significant difference of *OPG* expression measured (**Figure 3B** d13: p=0.4663, d20: p=0.0901) comparing monolayer PPF to monolayer PPF/PBMC co-cultures. The *RANKL*/*OPG* ratio of monolayer co-cultures stayed at the low level of monolayer PPF cultures at both time points (**Figure 4**).

#### 3.3.2 Gene Expression of Transwell Co-Cultures Acts More Like Baseline Tissue

Transwell PPF/PBMC co-cultures showed significantly higher expressions for *RANKL* (**Figure 3A**, d13: p<0.001, d20: p<0.001), *RANK* (**Figure 3C**, d13: p<0.001, d20: p<0.001), *MCSF* (**Figure 3D**, d13: p<0.001, d20: p<0.001) and *OCN* (**Figure 3E**, d13: p<0.001, d20: p<0.001) in comparison to monolayer co-cultures.

For *OPG* (**Figure 3B**, d13: p<0.001, d20: p<0.001), *TRAP* (**Figure 3G** d13: p= 0.2682, d20: p<0.001) and *CTSK* (**Figure 3F**, d13: p<0.001, d20: = 0.6833) significantly lower expressions were observed in transwell co-cultures compared to monolayer co-cultures.

The indicated baseline refers to relative mRNA expression of original periosteolytic tissue for each gene. It is noticeable that transwell co-cultures act closer to baseline conditions than monolayers considering especially *RANKL*, *OPG*, *OCN* and *CTSK*.

Regarding *RANKL*/*OPG* ratios, transwell co-cultures showed a significant higher expression, when compared to monolayers (**Figure 4**, d13: p= 0.0304, d20: p= 0.0455), resulting in a more osteoclastogenic environment. **Figure 4** shows that *RANKL*/*OPG* ratios of transwell cultures on both time points are very close to baseline conditions (**Figure 4**, d13: p=ns, d20: p= ns).

Baseline tissue corresponds to *in vivo* conditions, as periprosthetic tissue samples were used.

#### 3.3.3 Monocultures of PPFs in Transwell and Monolayer Show Similar Expression

Monocultures of PPFs showed about the same qPCR results in transwell as in monolayer cultures. In **Figure 3** only data from PPFs in monolayer cultures are shown to achieve a clearer graphical presentation.

### 3.4 RNA-Sequencing Data Analysis

#### 3.4.1 RNA-Sequencing Data Analysis Shows Equivalent Results for Orthopaedic and Dental Monolayer Cultures

**Figure 5:**
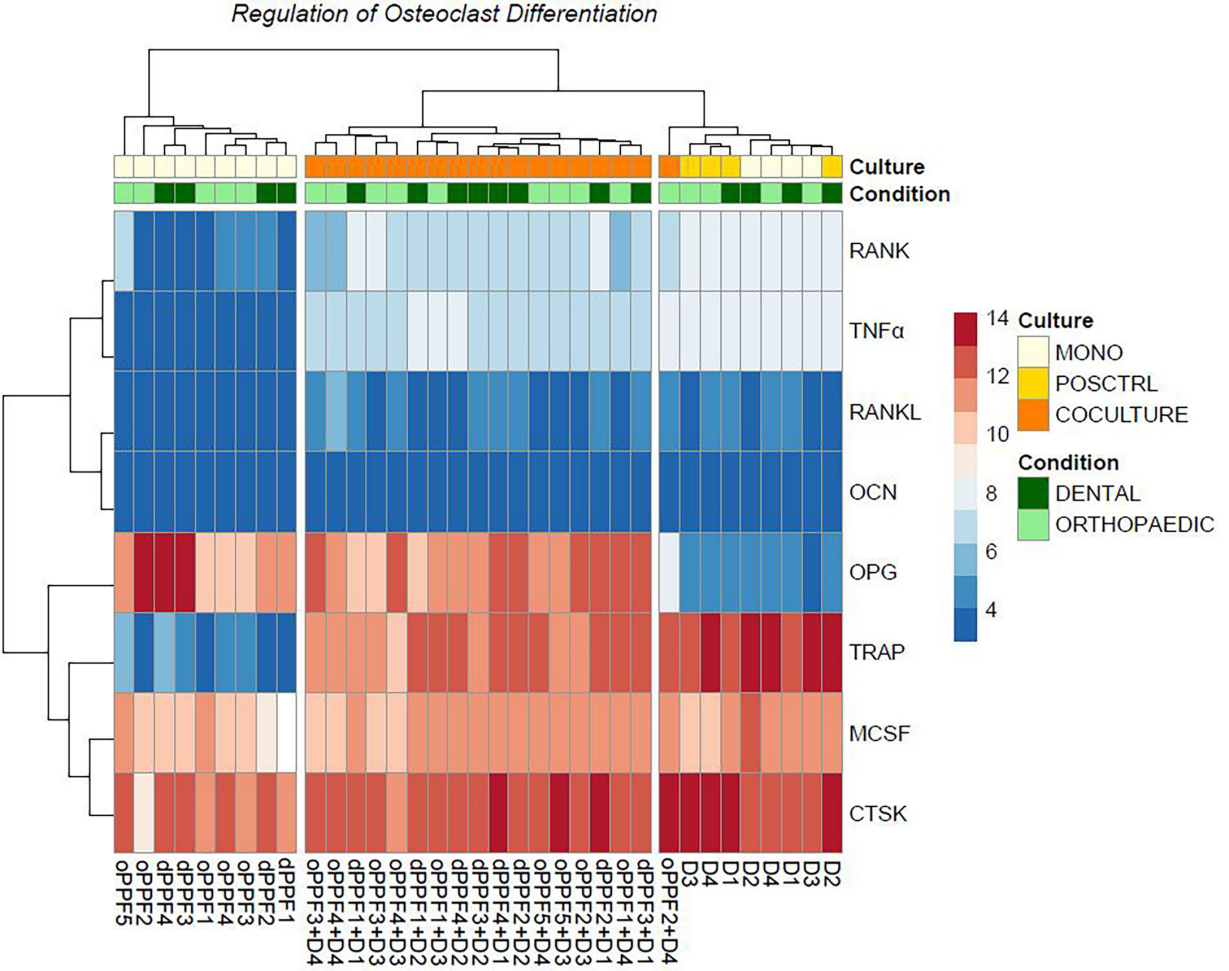
Heatmap of dental cultures on day 20. Ns, not significant.

Sequencing data results from day 20 are shown in **Figure 5** for dental and orthopaedic co-cultures, dental and orthopaedic monocultures of PPFs, stimulated and unstimulated PBMC cultures. This data is equivalent to the result of the qPCR and is independent of donors (donor of PBMCs =D). Most striking was the upregulation of OPG in PPF-monocultures and PPF/PBMC-co-cultures. Furthermore, an upregulation of TNFα, TRAP and RANK in PBMC-monocultures and PPF/PBMC-co-cultures was observed. The higher OPG expression in monolayer was therefore mainly caused by PPFs, whereas PBMCs were responsible for most of the TNFα expression.

#### 3.4.2 RNA-Sequencing Data: Top 50 Genes in Dental and Orthopaedic Monolayer Co-Cultures

**Figure 6:**
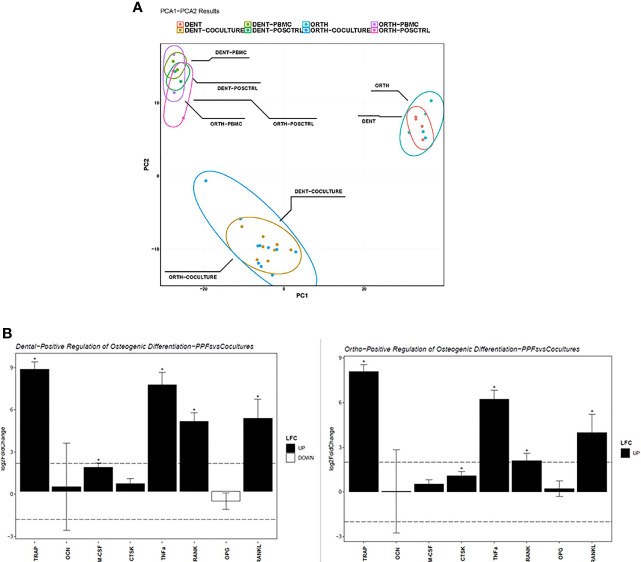
Sequencing analysis of dental and orthopaedic cultures on day 20. **(A)** Principal component analysis for co-cultures (PPF + PBMC), dental PPF (dPPF), orthopaedic PPF (oPPF), PBMC and positive control; **(B)** Positive regulation of Osteogenic Differentiation for dental (left) and orthopaedic (right) Co-cultures and PBMCs. Asterisks (*) indicate a p-value < 0.05 LFC, log2 fold change.

**Figure 6** shows the results of the RNA sequencing gene expression analysis of monolayers and co-cultures of dental and orthopaedic cell cultures all on day 20. There are 5 donors for orthopaedic cells and 4 donors for dental cells that are mono/co-cultured with PBMCs. The gene expression data were normalized by using the vst method. Even though the cells were derived from different origins, the PCA analysis showed similar gene expression patterns for each group: PBMCs, PPFs and co-cultures, with one single outlier: PPF2+ D4 co-culture (**Figure 6A**).

To validate the qPCR results, osteoclast differentiation regulator genes are filtered in each group from normalized gene expressions to check the variation. As an addition to the PCA clustering, osteoclast differentiation related genes also support the same clustering between each cell culture condition independent from the primary location of the cell. Furthermore, it shows the loss of *OPG* gene expression among the outlier PPF2+ D4 co-culture (**Figure 5**).

To understand how the osteoclast differentiation regulator gene expression varies between mono and co-culture in both dental and orthopaedic cell cultures, same genes are used from the qPCR results to filter DESeq2 analysis. Analysis performed in between mono and co-cultured samples gene expression for both dental and orthopaedic samples separately (**Figure 6B**). It is observed that for both of the dental and orthopaedic cell cultures, *TRAP, TNFα, RANK* and *RANKL* genes are upregulated significantly through co-culture of PPFs and PBMCs. Significance determined in DESeq2 analysis by 2 as log fold change cutoff and 0.05 as p adjusted value cutoff. It shows that PBMCs increases the osteoclast differentiation related gene expression in both dental and orthopaedic PPFs. Although different donors were used for PBMCs and PPFs, there is a strong overlap in the expression patterns of dental and orthopaedic cultures.

**Figure 7:**
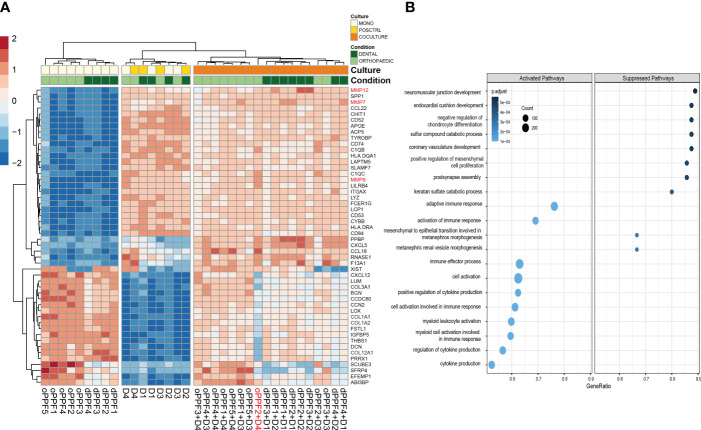
Top 50 genes and Gene Ontology Biological Pathway analysis on day 20. RNA-Sequencing data for orthopaedic and dental monolayer cultures on day 20: Top 50 genes that are differentially expressed between mono- and co-cultures, D (Donor)=PBMC **(A)**; Gene Ontology Biological Pathway analysis for mono- and co-cultures. Size of the dots are defined by the Gene Counts, color of the dots represent p-adjusted value **(B)**.

Top 50 genes that are differentially expressed between mono- and co-cultures show, besides the genes which are verified both in qPCR and RNA sequencing, there are other regulators such as *MMP*´s which are upregulated by co-cultures of PPFs and PBMCs (**Figure 7A**). MMP-13 and MMP-9 enhance osteoclastogenesis (36), whereas MMP-7 is accepted as one of the targets of RANKL to trigger osteolysis (37). The outlier PPF2+ D4 differs from the other co-cultured samples clearly observable in terms of gene expression.

Gene Ontology Biological Pathway analysis suggests that significant differentially expressed genes between mono- and co-cultured samples, independent from the driven location of the samples co-culturing PBMC and PPFs, show a suppression of several musculoskeletal related pathways, which are not directly connected to our experiments (**Figure 7B**). These results are however not supported yet by further data to prove the related gene expression results. It was observed on GO Pathways that pathways connected to immune responses might be activated within co-cultured samples (**Figure 7B**).

## 4 Discussion

Comparing dental and orthopaedic implant loosening processes overlaps in cellular components and their gene expression patterns, which refer to the same triggers and result in similar mechanisms, influencing the induction of osteoclastogenesis within peri-implant tissues of both types, have been discussed. Especially the (immune) regulatory effects of dental and orthopaedic PPFs, which play a major role in implant loosening, have been described in literature (10, 11, 14, 18, 23, 24, 38–40).

Monolayer co-cultures of PPFs of orthopaedic and dental origin expressed high levels of OPG over time resulting in an inhibition of osteolysis, even in the presence of MCSF and RANKL as overserved for PPFs from orthopaedic origin in earlier studies from this group (28) and for PPFs with dental origin co-cultured with PBMCs within this study. The high OPG expression in monolayer results in a low RANKL/OPG ratio, not representing the findings of original tissues where high RANKL/OPG ratios are usually described (11, 12, 31, 40). This effect, being due to the high OPG expression of PPFs in monolayer, can lead to a misinterpretation of results for dental and orthopaedic loosening, if monolayer cultures are used in evaluating processes of implant loosening. In monolayer cultures the immunomodulatory effect of PPFs is extremely efficiently blocking the osteoclastic activation of PBMCs due to an increase in OPG, with low RANKL, MCSF and TNFα expressions.

For transwell co-cultures, profound changes in gene expression, with a strong and significant decrease of OPG in PPFs of both origins (hundredfold in orthopaedic/twentyfold decrease in dental co-cultures) were observed. The RANKL/OPG ratios, which were rather low in monolayer co-cultures of both origins, were found to be significantly higher in transwell cultures and reached similar levels to original periprosthetic tissues. Several reviews state the importance of the RANKL/OPG pathway for the bone homeostasis, low levels of OPG lead to an activation of the osteoclastogenesis favoring bone resorption (41, 42). Elevated RANKL/OPG ratio were also found in peri-mucositis triggered by bacteria, peri-implantitis and gingival crevicular fluid of diseased peri-implant tissues (43–45). Increased RANKL/OPG ratios associated with bone resorption around dental implants were also shown in murine model (39). We hypothesized that the low OPG levels presented in the transwell co-culture system better reflect *in vivo* ratios of periprosthetic tissue than the increasing OPG expressions of the monolayer system. The transwell culture system allows improved cell-to-cell contacts favoring differentiation and intracellular signal pathways (46).

It has been shown that bone resorption cannot be equated with TRAP positivity and multinucleation in osteoclasts and PBMC cultures are capable of producing TRAP-positive cells without stimulation (3, 25, 28). In this study dentin chips were used as a gold standard for osteolysis. On dentin chips, all PBMC monocultures stimulated with MCSF/RANKL showed lacunae formation, whereas unstimulated PBMC cultures showed no lacunae formation. Cells from transwell cultures could be transferred to dentin chips after 21 days and showed about the same osteolytic activity as cells of monolayer cultures. Despite all similarities to original baseline tissue (corresponds to *in vivo* conditions), induction of osteoclastogenesis was absent in the co-cultures of the transwell system, where no resorption lacunae were found. Terminal osteoclastogenesis could not be induced by co-culturing dental PBMC/PPF due to similar reasons as described for orthopaedic co-cultures (28) and even an inhibition of osteoclastogenesis in stimulated co-cultures was observed.

Literature only shows complete differentiation of co-cultured fibroblasts and PBMCs in the presence of MCSF and RANKL, where the RANKL/OPG ratio had been higher in the first place, which might be an essential prerequisite. It may be postulated that the increased OPG expressed by orthopaedic and dental PPFs is sufficient to neutralize the elevated RANKL levels. Thus, dental and orthopaedic PPFs kept in monolayer can developed an osteoprotective effect that ultimately prevented active bone resorption (even when stimulated with MCSF and RANKL). This phenomenon of PPF inhibition of osteoclastogenesis occurred equally pronounced in co-cultures of dental or orthopaedic periprosthetic tissues within all donors. PPFs can significantly immunomodulate osteoclastic mechanisms and play an important role in dental and orthopaedic implant loosening. Comparing results from dental and orthopaedic gene expression pattern changes, many similar results were obtained for the groups of PBMCs, PPFs and co-cultures.

In our former study examining orthopaedic implant loosening, the same significant changes in gene expression of two major mediators of osteoclastogenesis were detected in transwell co-cultures compared to those in monolayer (28). The transwell co-cultures showed an extensive downregulation of OPG. We assumed, that the higher OPG expression in monolayer was mainly caused by PPFs, whereas PBMCs were responsible for most of the TNFα expression. The findings from this study make this assumption more reliable, as it can be shown that the origin of OPG is found in PPFs whereas PBMC seem to represent the origin of TNFα (**Figure 5**).

The role of TNFα might also play an important part, as TNFα is discussed as a direct mediator of osteoclastogenesis and a possible replacement for RANK-L. A RANK-L-independent formation of osteoclasts in the presence of MCSF and TNFα had been shown before in literature as described (28). Considering this alternative pathway, it may be assumed that expression of TNFα was insufficient in our studies to induce a RANK-L-independent osteoclast formation in the co-cultures. The *in vitro* conditions of transwell cultures mimic *in vivo* conditions to a certain content, but cannot reach the original tissue conditions. In the stimulated transwell co-cultures RANK-L and TNFα might have been insufficient to induce osteoclastogenesis.

In dental co-cultures the effects on gene expression obtained by transwell cultures are even more pronounced in some cases than in orthopaedic co-cultures. The expression of RANKL and MCSF increased significantly in transwell cultures of dental PPFs/PBMCs, whereas expression had only slightly increased in orthopaedic co-cultures (28). In transwells of dental origins, RANK was up-regulated in co-cultures compared to monolayer expression, whereas OPG as an opponent revealed an increased expression in monolayer co-cultures. This also corresponds to the findings of our previous study by Koehler et al. (28).

An overlap of expression within dental and orthopaedic co-cultures, PPFs and PBMCs was then further evaluated by RNA sequencing. The sequencing data reflected the results for the genes OPG, where PPF monocultures and PBMC/PPF co-cultures show rather high values in monolayer of all donors compared to PBMCs where low values for OPG are found in all donors. The OPG expression is therefore mainly driven by the PPFs of both origins. This further elucidates data from literature where PPF/PBMC co-cultures have been described to express high OPG levels (25, 38). According to sequencing and RT-PCR results RANK, TNFα and TRAP are upregulated in co-cultures and PBMCs of all donors compared to PPF monocultures. The RANK, TNFα and TRAP expression therefore seem mainly triggered by the PBMC input. When MCSF is added, TNFα-expressing PPFs cause increased osteoclast formation, the RANK receptor of osteoclast precursor cells is activated by RANKL, differentiation into osteoclasts increases their TRAP expression and thus contributes to endoprosthetic loosening. According to sequencing results the orthopaedic and dental samples show a strong overlap of processes. Dental and orthopaedic PPFs and their PBMC/PPF co-cultures showed broad agreement in principal component analysis and heat maps of differentially expressed genes. In particular, the upregulation of TRAP, TNFα and RANK associated with the downregulation of OPG leads to the osteoclastogenic differentiation in the co-cultures of both origins. This is, as to our knowledge, the first study showing the strong similarity between dental and orthopaedic peri-implant tissue cells, which supports the clinical thesis that similar immune responses play a crucial role in dental and orthopaedic implant loosening (23). Orthopaedic and dental implants represent foreign bodies to which the immunocompetent bone cells react and which, in case of mismatch, leads to bone resorption as evidenced by an increase in bone resorption markers. Therefore, the immune response by macrophages, foreign body giant cells, neutrophils etc. in orthopaedic and dental implant loosening might be similar and even might play a more decisive role than the biofilm theory. Albrektsson et al. hypothesized that bacterial pathogens arise only as a consequence to the foreign body reaction and thus, the immune response is the main reason for bone resorption (23). Considering osteoclasts as cells having an immune function besides the classical bone resorption activity, would furthermore open up the possibility for the hypothesis that degraded matrix components could leak out from the resorption lacuna, when the tight seal of this environment is loosened, and start a new resorption cycle (47).

In addition, recent studies using transcriptomic profiling show that periimplantitis is distinctly different from periodontitis in terms of molecular genetics. It has been shown that signaling pathways influencing the immune response are upregulated in peri-implantitis, whereas in periodontitis mainly bacterial response systems dominate (48, 49) and that higher RANKL/OPG ratios were measured, in particular, in peri-implant tissues (50). This reinforces our approach to compare dental and orthopaedic implants and suggests a common pathogenesis. To the best of our knowledge, this is the first study to have performed this direct comparison on a molecular level. On the one hand, this allows the transfer of scientific knowledge from one discipline to the other, although differences such as material, particle abrasion, biomechanics, etc. should be taken into account. On the other hand, understanding the primary pathogenesis opens up new therapy options in the field of regenerative medicine strategies such as platelet rich plasma, smart biomaterial-tissue interfaces or tissue-engineered Cell Sheets (51).

Especially the RANKL/OPG/RANK pathway, which is of interest in dental and orthopaedic implant osteolysis, needs to be further elucidated, as monolayer data here significantly deviate from transwell cultures, where more *in vivo* like results were obtained. Low OPG expression levels of periimplant tissues are stable, not dependent on time to revision surgery, endotoxin levels or other parameters (31). PPFs, which have been shown to express OPG, seem to play an important part in the remodelling of periprosthetic bone (52). Assuming that PPFs may be involved in osteoclastogenesis and bone resorption by the regulation of their expression leads to new understandings of implant loosening processes and might also change solution approaches.

Limitations of our study were the relatively small number of cases. However, sequencing data can prove that results in dental and orthopaedic peri-implant tissue cultures showed coherence with significant results. Another limitation of the study is the fact that PBMCs might induce donor specific immune reactions, which could influence the study results. To exclude any bias by donor-specific characteristics, two PBMC donors were used for each experiment and PPFs of each patient were co-cultivated with PBMCs of each donor separately. As the Gene Ontology Biological pathway analysis showed, co-cultures tended to increase immune responses. This might also be regarded as an unspecific finding due to donor reactions.

Morphological characterization of PPFs in transwell cultures had been performed by our group (34). Detailed histomorphological characteristics were not performed in the current study due to a limited number of cells per donor. Hartmann et al. found conglomerates of PPFs in frozen sections of transwell cultures forming a polylayer structure (34). This coincides with findings of Sabater et al. (27), showing that cultivating fibroblasts in transwell systems lead to an increased number of cells and higher cell mass compared to cultures on standard well bottoms, despite the smaller surface of the transwell membranes.

In summary, periprosthetic osteoclastogenesis may be a correlating immune process in orthopaedic and dental implant failure leading to comparable (immune) reactions with regard to osteoclast activation. One of the main players in osteoclastogenesis, OPG, which was found to be upregulated in monolayer co-cultures of both origins, experienced profound changes in gene expression with a twenty- to hundredfold decrease in the transwell culture system. In transwell co-cultures, RANKL/OPG ratios were significantly higher, reaching levels similar to those in the original periprosthetic tissue. The transwell cultures system may provide an *in vivo* like model for the exploration of orthopaedic and dental implant loosening. This study provides more indications that similar loosening processes occur in dental and orthopaedic implant failure and offers the transwell system as culture model to gain insight into both processes. Further studies are necessary to investigate the similarities of dental and orthopaedic implant loosening and further elucidate the immune processes regarding osteoclast activation of implant failure.

## Data Availability Statement

The datasets presented in this article are not readily available because of DSGVO/GDPR restrictions. Requests to access the datasets should be directed to the corresponding author.

## Ethics Statement

The studies involving human participants were reviewed and approved by LMU Ethic committee Munich. Written informed consent for participation was not required for this study in accordance with the national legislation and the institutional requirements.

## Author Contributions

SS, EH, MK, FB, JR, MS, EA, BH, SK, and SM-W: Study conception and design, analysis and interpretation of data, drafting of manuscript, and critical revision. SS, EH, MK, FB, JR, and SM-W: Acquisition of data. All authors have read and approved the final submitted manuscript.

## Funding

This work was supported by the “Deutsche Forschungsgemeinschaft” (DFG- MA 5158/1-1). 

## Conflict of Interest

The authors declare that the research was conducted in the absence of any commercial or financial relationships that could be construed as a potential conflict of interest.

## Publisher’s Note

All claims expressed in this article are solely those of the authors and do not necessarily represent those of their affiliated organizations, or those of the publisher, the editors and the reviewers. Any product that may be evaluated in this article, or claim that may be made by its manufacturer, is not guaranteed or endorsed by the publisher.

## Abbreviations

COX-2 Cyclooxygenase-2, CP Crossing Point, CTSK Cathepsin K, DIF Dental Interfaces, DMEM Dulbecco´s Minimal Essential Medium, EF1a Elongation factor 1alpha, Fig Figure, HE Hematoxylin and eosin, HKG Housekeeping gene, IF Interfaces (orthopaedics), MCSF Macrophage colony-stimulating factor, ML Monolayer, MMPs Matrix metallopeptidases, MNC multinucleated cell, OCN Osteocalcin, OPG Osteoprotegerin, PBMCs Peripheral blood mononuclear cells, PBS Phosphate buffered saline, PDLFs Periodontal ligaments fibroblasts, PGE2 Prostaglandin E2, PPFs Periprosthetic fibroblast-like cells, dPPFs Dental periprosthetic fibroblast-like cells, oPPFs Orthopaedic periprosthetic fibroblast-like cells, RANK Receptor activator of NF-κB, RANKL Receptor activator of NF-κB ligand, RT Room temperature, TNFα Tumor necrosis factor alpha, TRAP Tartrate resistant acid phosphatase, TW Transwell, α-MEM α-Minimal Essential Medium.

## References

[1] Perry MJ Mortuza FY Ponsford FM Elson CJ Atkins RM. Analysis of Cell Types and Mediator Production From Tissues Around Loosening Joint Implants. Br J Rheumatol (1995) 34(12):1127–34. doi: 10.1093/rheumatology/34.12.1127 8608353

[2] Sabokbar A Itonaga I Sun SG Kudo O Athanasou NA. Arthroplasty Membrane-Derived Fibroblasts Directly Induce Osteoclast Formation and Osteolysis in Aseptic Loosening. J Orthop Res (2005) 23(3):511–9. doi: 10.1016/j.orthres.2004.10.006 15885469

[3] Sabokbar A Kudo O Athanasou NA. Two Distinct Cellular Mechanisms of Osteoclast Formation and Bone Resorption in Periprosthetic Osteolysis. J Orthop Res (2003) 21(1):73–80. doi: 10.1016/S0736-0266(02)00106-7 12507582

[4] Konttinen YT Lappalainen R Laine P Kitti U Santavirta S Teronen O. Immunohistochemical Evaluation of Inflammatory Mediators in Failing Implants. Int J Periodontics Restorative Dent (2006) 26(2):135–41. 16642902

[5] Yucel-Lindberg T Olsson T Kawakami T. Signal Pathways Involved in the Regulation of Prostaglandin E Synthase-1 in Human Gingival Fibroblasts. Cell Signal (2006) 18(12):2131–42. doi: 10.1016/j.cellsig.2006.04.003 16766159

[6] Jiranek WA Machado M Jasty M Jevsevar D Wolfe HJ Goldring SR. Production of Cytokines Around Loosened Cemented Acetabular Components. Analysis With Immunohistochemical Techniques and in Situ Hybridization. J Bone Joint Surg Am (1993) 75(6):863–79. doi: 10.2106/00004623-199306000-00007 8314826

[7] Salcetti JM Moriarty JD Cooper LF Smith FW Collins JG Socransky SS. The Clinical, Microbial, and Host Response Characteristics of the Failing Implant. Int J Oral Maxillofac Implants (1997) 12(1):32–42. 9048452

[8] Wei X Zhang X Flick LM Drissi H Schwarz EM O'Keefe RJ. Titanium Particles Stimulate COX-2 Expression in Synovial Fibroblasts Through an Oxidative Stress-Induced, Calpain-Dependent, NF-kappaB Pathway. Am J Physiol Cell Physiol (2009) 297(2):C310–20. doi: 10.1152/ajpcell.00597.2008 PMC2724098 19494233

[9] Tsutsumi R Xie C Wei X Zhang M Zhang X Flick LM. PGE2 Signaling Through the EP4 Receptor on Fibroblasts Upregulates RANKL and Stimulates Osteolysis. J Bone Miner Res (2009) 24(10):1753–62. doi: 10.1359/jbmr.090412 PMC2743284 19419302

[10] Mandelin J Li TF Hukkanen M Liljestrom M Salo J Santavirta S. Interface Tissue Fibroblasts From Loose Total Hip Replacement Prosthesis Produce Receptor Activator of Nuclear Factor-KappaB Ligand, Osteoprotegerin, and Cathepsin K. J Rheumatol (2005) 32(4):713–20. 15801030

[11] Mandelin J Li TF Liljestrom M Kroon ME Hanemaaijer R Santavirta S. Imbalance of RANKL/RANK/OPG System in Interface Tissue in Loosening of Total Hip Replacement. J Bone Joint Surg Br (2003) 85(8):1196–201. doi: 10.1302/0301-620X.85B8.13311 14653607

[12] Crotti TN Smith MD Findlay DM Zreiqat H Ahern MJ Weedon H. Factors Regulating Osteoclast Formation in Human Tissues Adjacent to Peri-Implant Bone Loss: Expression of Receptor Activator NFkappaB, RANK Ligand and Osteoprotegerin. Biomaterials (2004) 25(4):565–73. doi: 10.1016/S0142-9612(03)00556-8 14607494

[13] Belibasakis GN Bostanci N. The RANKL-OPG System in Clinical Periodontology. J Clin Periodontol (2012) 39(3):239–48. doi: 10.1111/j.1600-051X.2011.01810.x 22092994

[14] Bordin S Flemmig TF Verardi S. Role of Fibroblast Populations in Peri-Implantitis. Int J Oral Maxillofac Implants (2009) 24(2):197–204. 19492634

[15] Wagner S Gollwitzer H Wernicke D Langer R Siebenrock KA Hofstetter W. Interface Membrane Fibroblasts Around Aseptically Loosened Endoprostheses Express MMP-13. J Orthop Res (2008) 26(2):143–52. doi: 10.1002/jor.20494 17853491

[16] Ma GF Ali A Verzijl N Hanemaaijer R TeKoppele J Konttinen YT. Increased Collagen Degradation Around Loosened Total Hip Replacement Implants. Arthritis rheumatism (2006) 54(9):2928–33. doi: 10.1002/art.22064 16948130

[17] Delaisse JM Andersen TL Engsig MT Henriksen K Troen T Blavier L. Matrix Metalloproteinases (MMP) and Cathepsin K Contribute Differently to Osteoclastic Activities. Microsc Res Tech (2003) 61(6):504–13. doi: 10.1002/jemt.10374 12879418

[18] Pap T Claus A Ohtsu S Hummel KM Schwartz P Drynda S. Osteoclast-Independent Bone Resorption by Fibroblast-Like Cells. Arthritis Res Ther (2003) 5(3):R163–73. doi: 10.1186/ar752 PMC165048 12723988

[19] Przekora A Ginalska G. Enhanced Differentiation of Osteoblastic Cells on Novel Chitosan/β-1,3-Glucan/Bioceramic Scaffolds for Bone Tissue Regeneration. BioMed Mater (2015) 10(1):015009. doi: 10.1088/1748-6041/10/1/015009 25586067

[20] Yamalik N Günday S Kilinc K Karabulut E Berker E Tözüm TF. Analysis of Cathepsin-K Levels in Biologic Fluids From Healthy or Diseased Natural Teeth and Dental Implants. Int J Oral Maxillofac Implants (2011) 26(5):991–7. 22010081

[21] Murata M Tatsumi J Kato Y Suda S Nunokawa Y Kobayashi Y. Osteocalcin, Deoxypyridinoline and Interleukin-1beta in Peri-Implant Crevicular Fluid of Patients With Peri-Implantitis. Clin Oral Implants Res (2002) 13(6):637–43. doi: 10.1034/j.1600-0501.2002.130610.x 12519339

[22] Maier GS Eberhardt C Strauch M Kafchitsas K Kurth AA. Is Tartrate-Resistant Acid Phosphatase 5b a Potent Bio-Marker for Late Stage Aseptic Implant Loosening? Int Orthop (2014) 38(12):2597–600. doi: 10.1007/s00264-014-2471-2 25082180

[23] Albrektsson T Becker W Coli P Jemt T Molne J Sennerby L. Bone Loss Around Oral and Orthopedic Implants: An Immunologically Based Condition. Clin Implant Dent Relat Res (2019) 21(4):786–95. doi: 10.1111/cid.12793 31134756

[24] Dickerson TJ Suzuki E Stanecki C Shin HS Qui H Adamopoulos IE. Rheumatoid and Pyrophosphate Arthritis Synovial Fibroblasts Induce Osteoclastogenesis Independently of RANKL, TNF and IL-6. J Autoimmun (2012) 39(4):369–76. doi: 10.1016/j.jaut.2012.06.001 PMC3593104 22867712

[25] de Vries TJ Schoenmaker T Wattanaroonwong N van den Hoonaard M Nieuwenhuijse A Beertsen W. Gingival Fibroblasts Are Better at Inhibiting Osteoclast Formation Than Periodontal Ligament Fibroblasts. J Cell Biochem (2006) 98(2):370–82. doi: 10.1002/jcb.20795 16440316

[26] Majety M Pradel LP Gies M Ries CH. Fibroblasts Influence Survival and Therapeutic Response in a 3D Co-Culture Model. PLoS One (2015) 10(6):e0127948. doi: 10.1371/journal.pone.0127948 26053043 PMC4460080

[27] Sabater D Fernández-López J-A Remesar X Alemany M. The Use of Transwells™ Improves the Rates of Differentiation and Growth of Cultured 3T3L1 Cells. Anal Bioanal Chem (2013) 405(16):5605–10. doi: 10.1007/s00216-013-6970-6 23604418

[28] Koehler MI Hartmann ES Schluessel S Beck F Redeker JI Schmitt B. Impact of Periprosthetic Fibroblast-Like Cells on Osteoclastogenesis in Co-Culture With Peripheral Blood Mononuclear Cells Varies Depending on Culture System. Int J Mol Sci (2019) 20(10). doi: 10.3390/ijms20102583 PMC6567687 31130703

[29] Lindhe J Meyle J. Peri-Implant Diseases: Consensus Report of the Sixth European Workshop on Periodontology. J Clin Periodontol (2008) 35(8 Suppl):282–5. doi: 10.1111/j.1600-051X.2008.01283.x 18724855

[30] Sanz M Chapple IL. Clinical Research on Peri-Implant Diseases: Consensus Report of Working Group 4. J Clin Periodontol (2012) 39(Suppl 12):202–6. doi: 10.1111/j.1600-051X.2011.01837.x 22533957

[31] Hartmann ES Kohler MI Huber F Redeker JI Schmitt B Schmitt-Sody M. Factors Regulating Bone Remodeling Processes in Aseptic Implant Loosening. J Orthop Res (2017) 35(2):248–57. doi: 10.1002/jor.23274 27116254

[32] Weinberg E Zeldich E Weinreb MM Moses O Nemcovsky C Weinreb M. Prostaglandin E2 Inhibits the Proliferation of Human Gingival Fibroblasts via the EP2 Receptor and Epac. J Cell Biochem (2009) 108(1):207–15. doi: 10.1002/jcb.22242 19582788

[33] Beck F Hartmann ES Koehler MI Redeker JI Schluessel S Schmitt B. Immobilization of Denosumab on Titanium Affects Osteoclastogenesis of Human Peripheral Blood Monocytes. Int J Mol Sci (2019) 20(5). doi: 10.3390/ijms20051002 PMC6429431 30813507

[34] Hartmann ES Schluessel S Köhler MI Beck F Redeker JI Summer B. Fibroblast-Like Cells Change Gene Expression of Bone Remodelling Markers in Transwell Cultures. Eur J Med Res (2020) 25(1):52. doi: 10.1186/s40001-020-00453-y 33121539 PMC7596965

[35] Yu G Wang LG Han Y He QY. Clusterprofiler: An R Package for Comparing Biological Themes Among Gene Clusters. Omics (2012) 16(5):284–7. doi: 10.1089/omi.2011.0118 PMC3339379 22455463

[36] Pivetta E Scapolan M Pecolo M Wassermann B Abu-Rumeileh I Balestreri L. MMP-13 Stimulates Osteoclast Differentiation and Activation in Tumour Breast Bone Metastases. Breast Cancer Res (2011) 13(5):R105. doi: 10.1186/bcr3047 22032644 PMC3262218

[37] Kessenbrock K Plaks V Werb Z. Matrix Metalloproteinases: Regulators of the Tumor Microenvironment. Cell (2010) 141(1):52–67. doi: 10.1016/j.cell.2010.03.015 20371345 PMC2862057

[38] Bloemen V Schoenmaker T de Vries TJ Everts V. Direct Cell-Cell Contact Between Periodontal Ligament Fibroblasts and Osteoclast Precursors Synergistically Increases the Expression of Genes Related to Osteoclastogenesis. J Cell Physiol (2010) 222(3):565–73. doi: 10.1002/jcp.21971 19927302

[39] Deng S Hu Y Zhou J Wang Y Wang Y Li S. TLR4 Mediates Alveolar Bone Resorption in Experimental Peri-Implantitis Through Regulation of CD45(+) Cell Infiltration, RANKL/OPG Ratio, and Inflammatory Cytokine Production. J Periodontol (2020) 91(5):671–82. doi: 10.1002/JPER.18-0748 PMC9930181 31489644

[40] Koulouvaris P Ly K Ivashkiv LB Bostrom MP Nestor BJ Sculco TP. Expression Profiling Reveals Alternative Macrophage Activation and Impaired Osteogenesis in Periprosthetic Osteolysis. J Orthop Res (2008) 26(1):106–16. doi: 10.1002/jor.20486 17729302

[41] Kim JM Lin C Stavre Z Greenblatt MB Shim JH. Osteoblast-Osteoclast Communication and Bone Homeostasis. Cells (2020) 9(9). doi: 10.3390/cells9092073 PMC7564526 32927921

[42] Carrillo-López N Martínez-Arias L Fernández-Villabrille S Ruiz-Torres MP Dusso A Cannata-Andía JB. Role of the RANK/RANKL/OPG and Wnt/β-Catenin Systems in CKD Bone and Cardiovascular Disorders. Calcif Tissue Int (2021) 108(4):439–51. doi: 10.1007/s00223-020-00803-2 33586001

[43] Shuto T Wachi T Shinohara Y Nikawa H Makihira S. Increase in Receptor Activator of Nuclear Factor κb Ligand/Osteoprotegerin Ratio in Peri-Implant Gingiva Exposed to Porphyromonas Gingivalis Lipopolysaccharide. J Dent Sci (2016) 11(1):8–16. doi: 10.1016/j.jds.2015.10.005 30894939 PMC6395150

[44] Theodoridis C Doulkeridou C Menexes G Vouros I. Comparison of RANKL and OPG Levels in Peri-Implant Crevicular Fluid Between Healthy and Diseased Peri-Implant Tissues. A Systematic Review and Meta-Analysis. Clin Oral Investig (2021). doi: 10.1007/s00784-021-04061-w 34264378

[45] Gürlek Ö Gümüş P Nile CJ Lappin DF Buduneli N. Biomarkers and Bacteria Around Implants and Natural Teeth in the Same Individuals. J Periodontol (2017) 88(8):752–61. doi: 10.1902/jop.2017.160751 28440740

[46] Knight E Przyborski S. Advances in 3D Cell Culture Technologies Enabling Tissue-Like Structures to Be Created In Vitro. J Anat (2015) 227(6):746–56. doi: 10.1111/joa.12257 PMC4694114 25411113

[47] Madel MB Ibáñez L Wakkach A de Vries TJ Teti A Apparailly F. Immune Function and Diversity of Osteoclasts in Normal and Pathological Conditions. Front Immunol (2019) 10:1408. doi: 10.3389/fimmu.2019.01408 31275328 PMC6594198

[48] Cho YD Kim PJ Kim HG Seol YJ Lee YM Ryoo HM. Transcriptome and Methylome Analysis of Periodontitis and Peri-Implantitis With Tobacco Use. Gene (2020) 727:144258. doi: 10.1016/j.gene.2019.144258 31759984

[49] Becker ST Beck-Broichsitter BE Graetz C Dörfer CE Wiltfang J Häsler R. Peri-Implantitis Versus Periodontitis: Functional Differences Indicated by Transcriptome Profiling. Clin Implant Dent Relat Res (2014) 16(3):401–11. doi: 10.1111/cid.12001 22967131

[50] Liu Y Liu Q Li Z Acharya A Chen D Chen Z. Long Non-Coding RNA and mRNA Expression Profiles in Peri-Implantitis vs Periodontitis. J Periodontal Res (2019) 55:343–53. doi: 10.1111/jre.12718 31853997

[51] Bijukumar DR McGeehan C Mathew MT. Regenerative Medicine Strategies in Biomedical Implants. Curr Osteoporos Rep (2018) 16(3):236–45. doi: 10.1007/s11914-018-0441-0 PMC6033057 29679306

[52] Koreny T Tunyogi-Csapóy M Gál I Vermes C Jacobs JJ Glant TT. The Role of Fibroblasts and Fibroblast-Derived Factors in Periprosthetic Osteolysis. Arthritis Rheum (2006) 54(10):3221–32. doi: 10.1002/art.22134 17009257

